# The effects of music combined to paired associative stimulation on motor-evoked potentials and alertness in spinal cord injury patients and healthy subjects

**DOI:** 10.1038/s41598-024-60984-w

**Published:** 2024-05-03

**Authors:** Kirsi Holopainen, Aleksi J. Sihvonen, Jaakko Kauramäki, Teppo Särkämö, Anastasia Shulga

**Affiliations:** 1 BioMag Laboratory, HUS Diagnostic Centre, Helsinki University Hospital, University of Helsinki and Aalto University School of Science, Helsinki, Finland; 2 Cognitive Brain Research Unit, Department of Psychology and Logopedics, Faculty of Medicine, University of Helsinki, Helsinki, Finland; 3 Centre of Excellence in Music, Mind, Body and Brain, University of Helsinki, Helsinki, Finland; 4 Clinical Neurosciences, Neurology, Faculty of Medicine, University of Helsinki, Helsinki, Finland; 5 Department of Neurology, Neurocenter, Helsinki University Hospital, Helsinki, Finland; 6 Department of Physical and Rehabilitation Medicine, Helsinki University Hospital and University of Helsinki, Helsinki, Finland

**Keywords:** Neuroscience, Neurology

## Abstract

Paired associative stimulation (PAS) consisting of high-intensity transcranial magnetic stimulation (TMS) and high-frequency peripheral nerve stimulation (known as high-PAS) induces plastic changes and improves motor performance in patients with incomplete spinal cord injury (SCI). Listening to music during PAS may potentially improve mood and arousal and facilitate PAS-induced neuroplasticity via auditory-motor coupling, but the effects have not been explored. This pilot study aimed to determine if the effect of high-PAS on motor-evoked potentials (MEPs) and subjective alertness can be augmented with music. Ten healthy subjects and nine SCI patients received three high-PAS sessions in randomized order (PAS only, PAS with music synchronized to TMS, PAS with self-selected music). MEPs were measured before (PRE), after (POST), 30 min (POST30), and 60 min (POST60) after stimulation. Alertness was evaluated with a questionnaire. In healthy subjects, MEPs increased at POST in all sessions and remained higher at POST60 in PAS with synchronized music compared with the other sessions. There was no difference in alertness. In SCI patients, MEPs increased at POST and POST30 in PAS only but not in other sessions, whereas alertness was higher in PAS with self-selected music. More research is needed to determine the potential clinical effects of using music during high-PAS.

## Introduction

Paired associative stimulation (PAS) is a non-invasive stimulation method that combines transcranial magnetic stimulation (TMS) with peripheral electrical nerve stimulation (PNS)^1^. PAS has been used to enhance spinal^2–4^ and cortical plasticity^5^. TMS induces neuronal activity in motor cortical circuits and orthodromic activation of the pyramidal tract, and PNS triggers antidromic activation that starts in peripheral somatosensory and lower motor neurons^6^. The TMS and PNS stimuli can be set to converge at the cortical^1,5^ or spinal level^6,7^. Synchronous activation of pre- and postsynaptic neurons is thought to enhance synaptic strength via long-term potentiation (LTP)-like mechanisms^5,8^. In healthy individuals, PAS can induce both potentiation and inhibition depending on frequency, intensity, and timing of the stimuli^1,9,10^. In PAS experiments, the interstimulus interval (ISI) between TMS and PNS has been shown in many cases to determine the polarity of the responses^1,4^. Motor-evoked potentials (MEPs) are responses to TMS recorded from muscles, and their average amplitude can increase or decrease after PAS, indicating facilitation or inhibition, respectively^1^.

A modified version of PAS, called high-PAS ^11^, uses navigated TMS (nTMS) with high intensity (100% stimulator output) and PNS with high frequency (100 Hz). This method can successfully enhance motor performance in patients with incomplete spinal cord injury (SCI)^11–13^. Over 20 patients with incomplete SCI with a wide range of injury severity, age, and time since injury have received high-PAS and have exhibited improved motor function and recovery^11^. In contrast to conventional PAS protocols, which aim to target the cortical level through activation of sensory fibres^5,9,10^, high-PAS specifically aims to activate motor fibres and induce therapeutically useful plastic response at the spinal level of the corticospinal tract^2,11^. In high-PAS, the timing of cortical and peripheral stimulations is set to induce multiple simultaneous orthodromic and antidromic activations in the corticospinal tract and at the spinal level to increase spinal excitability and connectivity^11^. This phenomenon occurs because the interactions leading to LTP-like effects overcome their long-term depression (LTD)-like counterparts^14^. It is possible that high-PAS improves corticospinal conduction both by rerouting interneurons^15^ and directly strengthening weakened synaptic connections between upper and lower motor neurons^1^, because the corticospinal fibres terminate both on interneurons and on alpha and gamma motoneurons of the spinal cord^16^. In contrast to conventional PAS protocols, which require precisely defined parameters, high-PAS tolerates small errors in mapping and at least ± 10 ms errors in ISI determination, which makes it more feasible for clinical use^11,17^. Our group has previously demonstrated that 100-Hz PNS is more efficient than lower (25 or 50 Hz) or higher (200 or 400 Hz) PNS frequencies in high-PAS^18,19^.

In previous studies on high-PAS, patients or healthy subjects were not allowed to engage in any external leisure activities, such as talking, watching TV, or using their smartphone during the high-PAS session. This is based on conventional PAS studies, which have shown that attention plays an important role in PAS-induced plasticity^20^. When using high-PAS as therapy, patients receive up to six 20-min high-PAS sessions in row, which is 2 h of stimulation. Many patients find such long stimulation sessions tiring and boring or have difficulty staying awake and focusing on motor imagery or pre-activation of the muscle of interest, which are often included in PAS sessions.^11^ Sleeping or drowsiness should be avoided, as clear reduction in alertness can change functional connectivity and motor thresholds and make the therapeutic response unpredictable^20^.

Music is a particularly powerful tool for regulating mood and arousal and could potentially be used in high-PAS sessions. Music can evoke strong emotions, induce pleasure, enhance mood^21^, and improve performance on tasks involving focused or sustained attention, either by listening to or using music within a procedure to modulate mood and motivation^21–24^. This effect seems to be linked to increased activation in the frontoparietal attention network induced by music^25,26^, coupled with engagement of the mesolimbic dopaminergic reward system, which mediates music-induced pleasure^21,27^. Music also effectively directs attention to itself, away from other stimuli or internal experiences, which has been linked to its analgesic effects in reducing pain^26,28,29^, which is also relevant for any PAS protocols. In a recent high-PAS case study, one SCI patient who had serious difficulties staying awake during long stimulation sessions listened to music of his own choice and this did not prevent the therapeutic effect of high-PAS (i.e. the patient’s motor function improved despite the music)^12^. Together, these findings suggest that music may be applicable to support mood and arousal during PAS.

In addition to its cognitive-affective effects, music also has another key characteristic that may potentially support PAS. Music has a regular tempo or pulse known as beat, which enables listeners to synchronize their body movements (e.g., tap feet, clap hands, or dance) to a musical piece. Neuroimaging studies have conclusively shown that musical beat processing engages a large network of auditory and motor regions, including bilateral superior temporal cortices, supplementary motor area, putamen, and cerebellum^28,29^ and that motor regions are activated even when listening to musical rhythm without movement^30,31^. Mediated by the close interaction between the auditory and motor systems (referred to as auditory-motor coupling), musical beat is tracked and anticipated by repeated movement-like processes in the motor system that are coordinated through rapid bidirectional communication with the auditory cortex^32^. Together with the reward system, the auditory-motor regions form an internal representation of the musical beat, entrain movements to it, and create a positive emotional experience and desire to move to music^33^.

There are many auditory-related mechanisms that can promote reticulospinal and cortico-reticular connections. Although the main function of reticulospinal tract is to control posture, locomotion, and muscle tone, it may also have a role in hand function^34^. The acoustic startle reflex is a low-latency reflex, a muscle response elicited by a sudden and intense acoustic stimulus. Acoustic startle cannot be detected if ventral regions of the nucleus reticularis pontis caudalis are not functional^35^. These regions contain cell bodies that give rise to the reticulospinal tract. Input to the reticulospinal tracts comes from many areas of the brain, including the motor areas of the cerebral cortex^36^. Studies have demonstrated that pairing auditory stimulus with TMS increases motor system excitability measured by MEPs^37^ and with electrical stimulation modifies spike timing-dependent plasticity in reticulospinal circuits^38^. TMS also activates cortico-reticular pathways in primates. TMS evoked both short- and long-latency responses, and the latter was associated with click sound made by coil discharge.^39^.

Importantly, auditory-motor coupling also occurs when the motor cortex is stimulated externally. Previous TMS studies have shown that the presentation of music during TMS applied to the motor cortex enhances MEP amplitude recorded from muscles compared to TMS only^40^, indicating that music can enhance corticospinal excitability. Interestingly, this effect seems to be particularly strong for music with a strong metrical rhythm or high groove beat^41,42^ and after subjects have learned the melody^43^. Emotions evoked by music can also mediate the enhancing effect.^44^. Clinically, playing-based music rehabilitation in chronic stroke patients can enhance MEP amplitude recorded from the lesioned hemisphere^45,46^, indicating that music combined with motor exercise can enhance plasticity within the affected motor cortex. Previous studies have shown higher PAS-induced MEP amplitudes in musicians^47,48^, but the effects of concurrent music stimulation during high-PAS or any other PAS protocol have thus far not been explored.

The rationale of the study arose from both practical clinical challenge of how to keep up some SCI patients’ mood and arousal during long stimulation protocol which has otherwise shown therapeutic potential, and from the need to investigate whether such powerful additional external stimulus as music would contribute to or hinder this therapeutic effect. The purpose of this pilot study was to explore the effects of high-PAS combined with music on MEPs (primary outcome) recorded at pre-stimulation and three different post-stimulation time points and on subjective arousal and comfort (secondary outcomes) during high-PAS in healthy subjects and in SCI patients. In this study, we did not aim to compare healthy subjects and SCI patients, but to separately report the results of both groups. Building on the rationale that music during high-PAS may potentially improve mood and arousal and facilitate PAS-induced associative plasticity via auditory-motor coupling, we utilized both self-selected music and music that was temporally synchronized to the timing of the TMS pulses and compared these with high-PAS without additional stimulation. We hypothesized that self-selected music would specifically improve subjective arousal and comfort during high-PAS and that synchronized music would specifically enhance PAS-induced MEPs.

## Methods

### Participants and study design

Participants were 10 healthy subjects (2 males and 8 females, mean age 26.0 SD ± 5.31 years) and 9 patients with incomplete SCI (6 males and 3 females, mean age 58.44 ± 8.38 years). Eight of nine SCI patients had previously participated in high-PAS experiments. Time since injury was 7.4 ± 3.8 years. Time since last active PAS session was at least 5 months. Eight patients had grade D on ASIA impairment scale (AIS)^37^ and one patient had grade B. More detailed information on the patients is presented in Table 1. Patient 6 was excluded from MEP data because of unclear and unreliable MEP recordings due to strong spasticity and resting motor threshold (RMT) fluctuation. The patient’s answers to questionnaires were still included. Each participant provided written consent before participation. The study was approved by the Helsinki University Hospital Regional Committee on Medical Research Ethics. Exclusion criteria for healthy subjects were any brain pathology, implanted devices, regular medication, neurological diseases, cardiac diseases, psychiatric diseases, and pregnancy.

**Table 1:** Information on SCI patients.

ID	Age	Gender	Year of injury	Level of injury	AIS grade	CNS-active continuous medicines, total daily dose
1	49	M	2020	C3	D	Baclofen 60 mg, pregabalin 100–200 mg
2	53	F	2014	C6	D	Gabapentin 2700 mg, duloxetine 90 mg
3	60	M	2011	C2	D	Amitriptyline 30 mg
4	66	M	2022	C2	D	–
5	70	F	2018	C1	D	Buprenorphine 5 µg/h, duloxetine 90 mg
6	66	F	2013	C1	D	tizanidine 6 mg
7	56	M	2016	C2	D	Pregabalin 225 mg, baclofen 50 mg
8	45	M	2011	C7	B	Tramadol 100 mg + 50 mg, baclofen 25 mg, clonazepam 2 mg
9	61	M	2015	C5	D	Baclofen 60 mg, tizanidine 24 mg, pregabalin 225 mg

CNS-active = central nervous system active.

**Figure 1:**
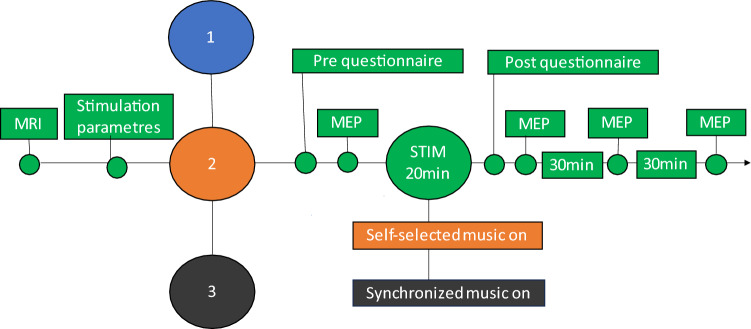
Study design and structure of one stimulation session. Numbers 1–3 describe different sessions. Green colour indicates events common for all three sessions. Blue—PAS only, orange—PAS with self-selected music, black—PAS with synchronized music.

The experiment was performed at the BioMag laboratory at Helsinki University Hospital (Helsinki, Finland). All participants visited the laboratory three times for high-PAS. Sessions were PAS only (later PAS or PAS only), PAS with subject’s self-selected favourite music (MUSIC), and PAS with music synchronized to TMS pulses (SYNC). Using a within-subject design, every participant received three sessions in randomized order. Time between sessions was minimum 7 days and on average 9.3 ± 4.1 days. Study design and the structure of one stimulation session are presented in Fig. 1. A more detailed description of the sessions is provided below. Structural brain MRI was taken from each participant for TMS navigation and safety. Eight of nineteen participants had stimulation parameters determined on the same day as the first PAS session, with at least a 30-min resting period in between.

### Cortical mapping and MEP recordings

The target area (hotspot) for the muscle of interest in the primary motor cortex (M1) was determined using individual MRI images and electromyographic (EMG) signal and saved for stimulation sessions. MEPs were recorded using Nexstim eXimia magnetic stimulator (Helsinki, Finland) with an EMG device integrated to an eXimia magnetic stimulator (band-pass filter 10–500 Hz, sampling rate 3 kHz) and surface electrodes (Neuroline 720, AMBU A/S, Ballerup, Denmark) placed over the target muscle. The target muscle was the left abductor hallucis (AH) in healthy subjects and the abductor pollicis brevis (APB) of the dominant hand (right n = 7, left n = 2) in SCI patients. AH was chosen as a target muscle for healthy subjects because it was successfully stimulated in our earlier experiments ^18,19,49,50^ and in our experience there is less intrinsic MEP amplitude variability in feet than in hand muscles in healthy subjects. However, using the AH was not feasible for SCI patients due to high resting motor threshold (RMT) or low functionality of the feet muscles (see Discussion).

For definition of MEP latency (required for determination of interstimulus interval (ISI) between TMS and PNS) and for MEP recordings during experiments, TMS intensity was set individually at 120% of resting motor threshold (RMT). RMT of the hotspot was determined as the lowest TMS intensity that produced MEPs of at least 50 µV in a minimum of 5/10 attempts^51^. For MEP latency determination, 15 MEPs were recorded, and their mean value calculated. For experimental sessions, MEP amplitudes were assessed before (PRE), immediately after (POST), 30 min after (POST30), and 60 min (POST60) after the stimulation. At each assessment, 30 TMS pulses were given every 3.3 s, and the mean amplitude of 30 MEPs was calculated. Individual MEPs that had > 50 µV EMG artifact spikes in 150-ms time period before the pulse were removed.

### Peripheral nerve parameters and stimulation

PNS was delivered with Dantec Keypoint® (Natus Medical Incorporated, California, USA) as trains of six 1-ms square pulses at 100 Hz. Stimulation was applied to the tibial nerve behind the left malleolus in healthy subjects and to the median nerve in the middle of the palmar side of the wrist in SCI patients. Stimulation intensity was set individually to the lowest intensity where F-responses were detectable when recorded with 1-ms pulses, as previously described^52^. For F-latency determination required for ISI calculation, 0.2-ms pulses at supramaximal intensity were applied and F-responses recorded from the AH for healthy subjects or APB for patients, and minimum latency was determined. For healthy subjects, a local anaesthetic (5% lidocaine/prilocaine [EMLA]) was used to prevent the pricking skin sensation associated with the stimulation in the sensitive area of the ankle. EMLA penetrates 3–5 mm into the skin and thus does not affect the conductivity of the tibial nerve^53^. Local anaesthetic in the wrist was not needed.

### Paired associative stimulation

PAS sequences (240 pulses) were triggered through Presentation® software (Neurobehavioral Systems Inc., Albany, USA) once every 5 s (20 min in total). A more detailed description of the high-PAS protocol can be found in previous articles^11–13^. During PAS, navigated TMS (eXimia magnetic stimulator, Nexstim Ltd, Helsinki, Finland) was delivered using single pulses at 100% of the maximum stimulator output, corresponding to 172 V/m ± 2 measured 25 mm below the figure-of-eight coil (Nexstim NBS manual) to AH or APB hotspot determined as described above. ISI between TMS pulse and the first train of the PNS pulse was calculated individually by the formula [F latency—MEP latency] to make the stimuli coincide at the spinal cord level as described previously^52^. F latency and MEP latency determination is described above. PNS was delivered by Dantec Keypoint with individualized settings described above. All participants were instructed to think about moving the muscle of interest (motor imagery).

### Description of PAS sessions and music

In PAS only sessions, participants received 20 min of PAS in silence using earplugs and were not allowed to speak during the stimulation. In MUSIC sessions, participants listened to their self-selected favourite music during 20 min of high-PAS using their own mobile device and earphones. After the stimulation, participants gave a list of the songs they listened to and described the genre(s) of the music. No restrictions were placed on the style, tempo, rhythm, or volume of the music. Speaking during stimulation was not allowed in this session.

**Figure 2:**
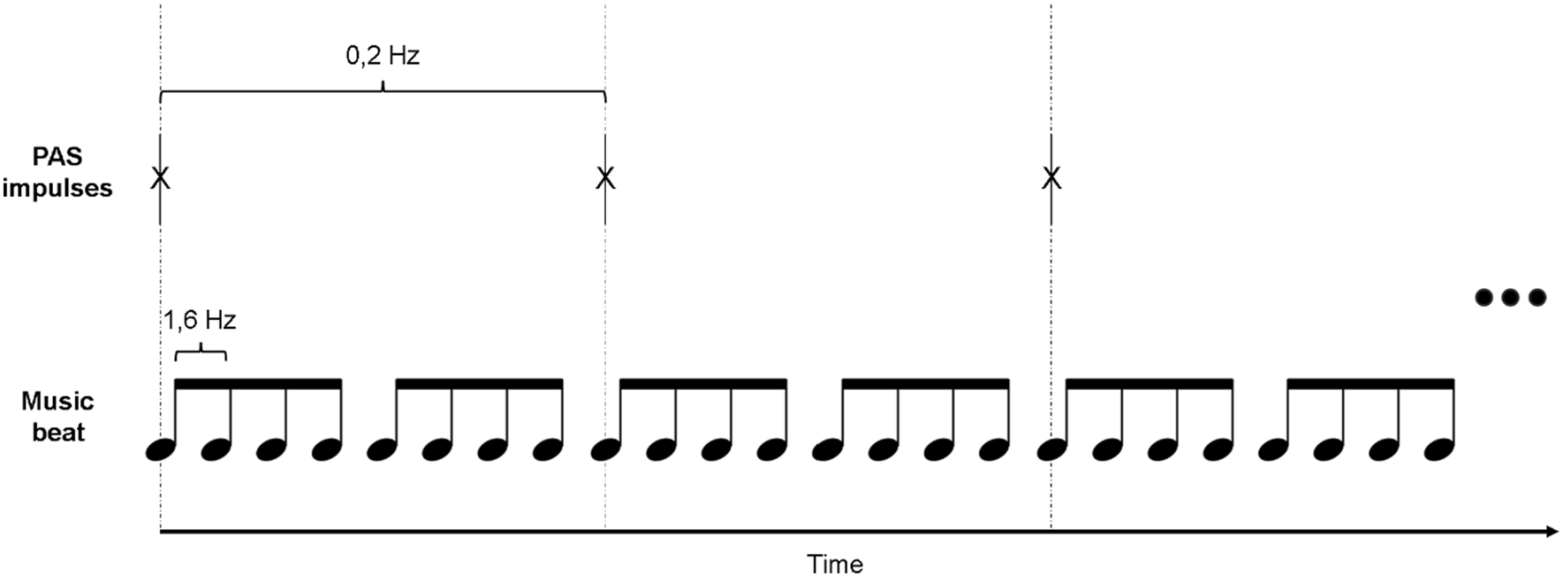
Schematic illustration of PAS pulse and music beat synchronization. The PAS pulse was simultaneous with every eighth beat of the music.

In SYNC sessions, participants listened to music synchronized with TMS pulses during 20 min of high-PAS. For this, a novel uplifting and energetic electronic funk music piece with a strong drumbeat was composed by author AJS using Apple GarageBand (Supplementary information 3, MP3 file). The music piece was 20 min long and rhythmically (96 bpm) synchronized to the TMS pulses (Fig. 2). Music and TMS pulse synchronization was performed using a modified Presentation experiment, where the music piece was started prior to the stimulation sequence start. Audio track start was offset so that the strong beat co-occurred with the TMS auditory click throughout the experiment. The relative synchrony of the TMS stimulation and music was verified by an external audio recorder (Zoom H6n, Zoom Corporation, Tokyo, Japan) with one external microphone placed next to the TMS coil and Zoom H6n stereo microphone recording the auditory track. Initially the onset of the audio track and TMS pulse click train was perceptually adjusted to be simultaneous; this way the TMS pulse preceded the musical beat by 56 ± 2 ms at the beginning of the song. Due to small differences in the audio playback speed and Presentation software and computer clock controlling the TMS sequences, the difference diminished throughout the recording with a linear 23.6-ms/10-min pace. At the end of the full 20-min song, the TMS pulses and musical beats were closer in time (within 10 ms). While the maximal difference in alignment was above the perceptual threshold of about 20 ms for isochronous sound sequence^54^ due to slowly changing asynchrony and psychoacoustical forward masking (strong TMS-elicited click preceding), this likely did not elicit a feeling of misalignment of the TMS pulse and musical beat for the subjects.

### Alertness and comfort

Information on the experienced alertness level was collected with a short questionnaire before the stimulation. After the stimulation, alertness and comfort during the stimulation were assessed with same type of questionnaire. Answers to the five-point scaled questionnaire were provided as emojis. For example, when asking about alertness, the answer “very alert” was a happy emoji and “very tired” a sad emoji (see Supplementary information 2). In addition to the questionnaire answers, verbal feedback given by the participants was recorded.

### Statistical analysis

All data from MEP recordings and questionnaires were analysed with IBM SPSS statistics 28.0. The results from healthy subjects and SCI patients were analysed separately. Non-parametric tests were used since the data were non-normally distributed in Kolmogorov–Smirnov and Shapiro–Wilk tests of normality. MEP data from the four time points (PRE, POST, POST30, POST60) were first analysed separately for the three sessions using Friedman’s two-way ANOVA for related samples, followed by post hoc testing with the Dunn-Bonferroni test. In post hoc testing, we performed three pair-wise comparisons (PRE vs. POST, PRE vs. POST30, and PRE vs. POST60). Accordingly, the alpha level was set to 0.05/3 = 0.0167 (Bonferroni correction) to control for multiple comparisons. We next used these same tests to compare the three sessions to each other regarding the baseline (PRE) MEP amplitudes and their percentual change scores from baseline to the three post-stimulation time points (POST/PRE*100, POST30/PRE*100, POST60/PRE*100). With the questionnaire data, we used the Friedman and Dunn-Bonferroni tests to compare the three sessions for arousal scores before and during the PAS stimulation and comfort scores during the stimulation.

### Ethical approval

The studies involving human participants were reviewed and approved by the Helsinki University Hospital Regional Committee on Medical Research Ethics. The entire study was performed in accordance with the Declaration of Helsinki. All participants provided their written informed consent to participate in this study.

## Results

### Changes in MEP amplitudes in healthy subjects

In within-session Friedman’s tests, there were significant changes in MEP amplitudes across the four measurement points (PRE, POST, POST30, POST60) in all three sessions [PAS only: Χ^2^(3) = 14.88, *p* = 0.002; PAS with self-selected music: Χ^2^(3) = 16.68, *p* = 0.001; PAS with synchronized music: Χ^2^(3) = 17.40, *p* = 0.001]. Post hoc testing with the Dunn-Bonferroni tests showed that MEP amplitudes increased significantly from PRE to POST in all three sessions (PAS only: *p* < 0.001, PAS with self-selected music: *p* < 0.001, PAS with synchronized music: *p* < 0.001) and from PRE to POST60 in PAS only (*p* = 0.015) and in PAS with synchronized music (*p* = 0.001). The changes from PRE to POST30 did not reach the significance threshold (PAS only: *p* = 0.038, PAS with self-selected music: *p* = 0.083, PAS with synchronized music: *p* = 0.057).

**Figure 3:**
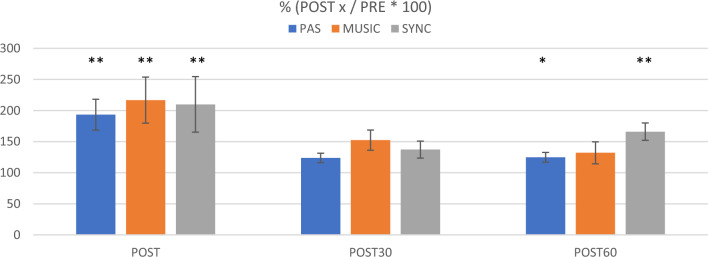
Healthy subject MEP amplitude change vs PRE (100%) and standard errors. **p ≤ 0.005 *p ≤ 0.0167.

**Figure 4:**
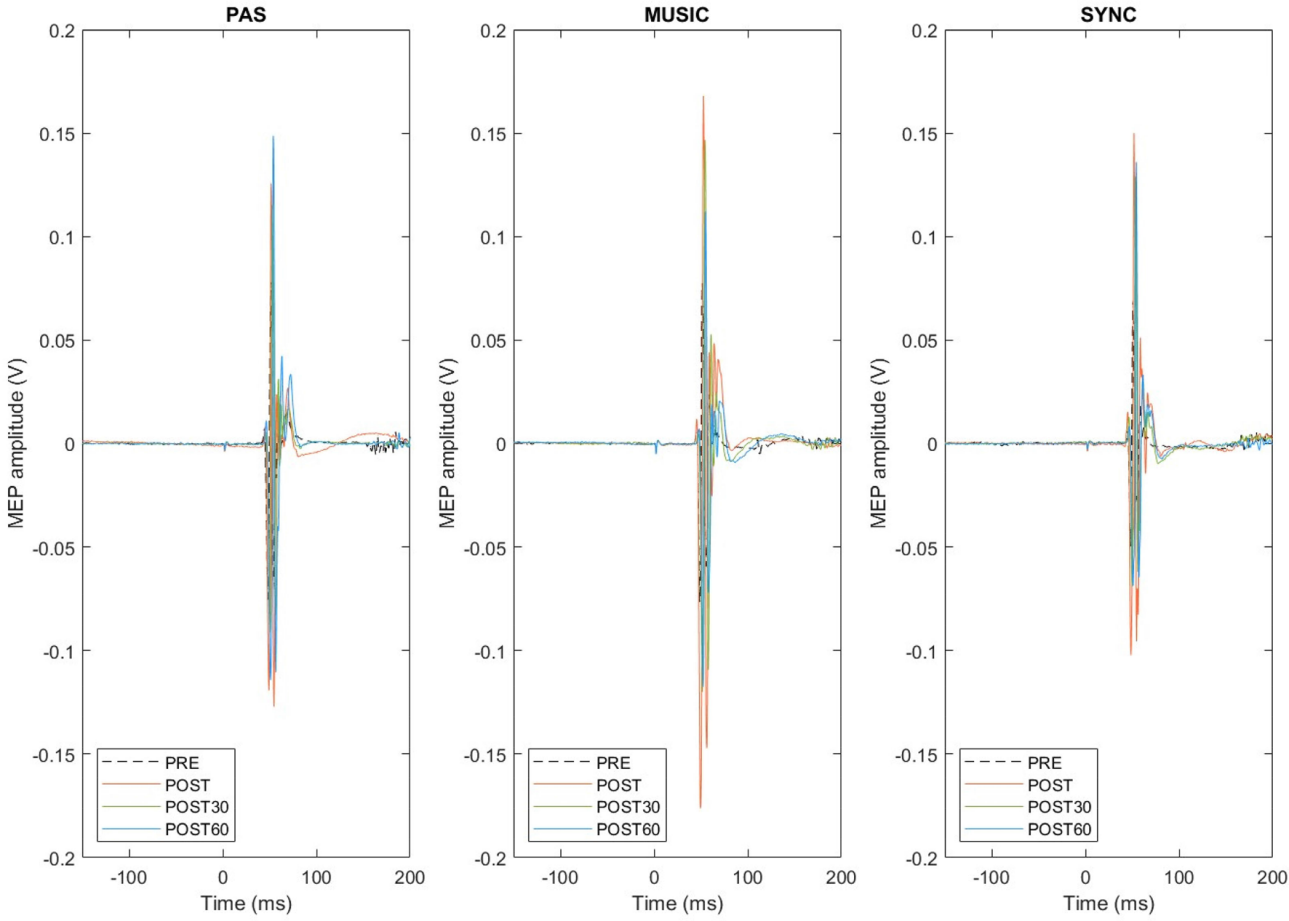
Representative MEP traces from one healthy subject (number 9). Each picture presents one session, each trace represents an average of 30 MEPs. Detrend function was used to bring pre stimulation EMG baseline to 0mV and 50Hz noise was filtered with a notch filter. Number 0 on x-axis (time) describes the time of given TMS pulse.

In between-session Friedman’s tests, the MEP amplitudes did not differ significantly between the three sessions at baseline (PRE) [Χ^2^(2) = 0.80, *p* = 0.670], from PRE to POST [Χ^2^(2) = 2.60, *p* = 0.273], or from PRE to POST30 [Χ^2^(2) = 2.60, *p* = 0.273], but there was a significant difference between the sessions from PRE to POST60 [Χ^2^(2) = 9.60, *p* = 0.008]. Post hoc testing (Dunn-Bonferroni) showed that the MEP amplitudes increased from PRE to POST60 more in PAS with synchronized music than in PAS only (*p* = 0.007) or PAS with self-selected music (*p* = 0.007). Together, these results indicate that in healthy subjects all three PAS sessions yielded a marked increase in MEPs immediately after stimulation, but the effects of the stimulation remained stronger 60 min after the stimulation when music synchronized to TMS pulses was used (Fig. 3). Original MEP traces from one representative subject (Subject 9) are presented in Fig. 4. In this subject, highest peak can be seen in POST60 (blue) in PAS and especially in SYNC compared to PRE level (black, dotted line), but in contrast in MUSIC highest peak is in POST (orange). MEP results of all subjects as absolute values (µV) and percents (% POSTx/PRE*100) are presented in Supplementary information 1a.

### Subjective alertness and comfort in healthy subjects

Subjective alertness and comfort scores of healthy subjects are shown in Table 2. In Friedman’s tests, there were no significant differences between the three sessions in alertness before the stimulation [Χ^2^(2) = 1.18, *p* = 0.554], in alertness [Χ^2^(2) = 4.33, *p* = 0.115], or comfort [Χ^2^(2) = 3.26, *p* = 0.196] during the stimulation.

**Table 2:** Healthy subjects (n=10) averaged alertness and feelings on scale 1–5 (alertness: 1 = very alert, 5 = very tired, feelings: 1 = very comfortable, 5 = very uncomfortable).

Session	Alertness PRE	Alertness DURING	Feelings DURING
PAS only	2.5 ± 0.3	2.8 ± 0.2	2.3 ± 0.2
PAS + own music	2.2 ± 0.2	2.5 ± 0.3	2.8 ± 0.2
PAS + sync music	2.4 ± 0.3	2.4 ± 0.3	2.6 ± 0.2

In the verbal feedback given by the healthy subjects after stimulation, 6/10 subjects preferred synchronized music over no music, either because it made the stimulation feel less uncomfortable or helped pass the time, whereas 3/10 subjects felt it was irritating. No negative feedback was given in the session where subjects had listened to self-selected music. When comparing feedback to MEP results there was no correlation; synchronized music also increased MEPs among the subjects who found it irritating.

### Changes in MEP amplitudes in SCI patients

**Figure 5:**
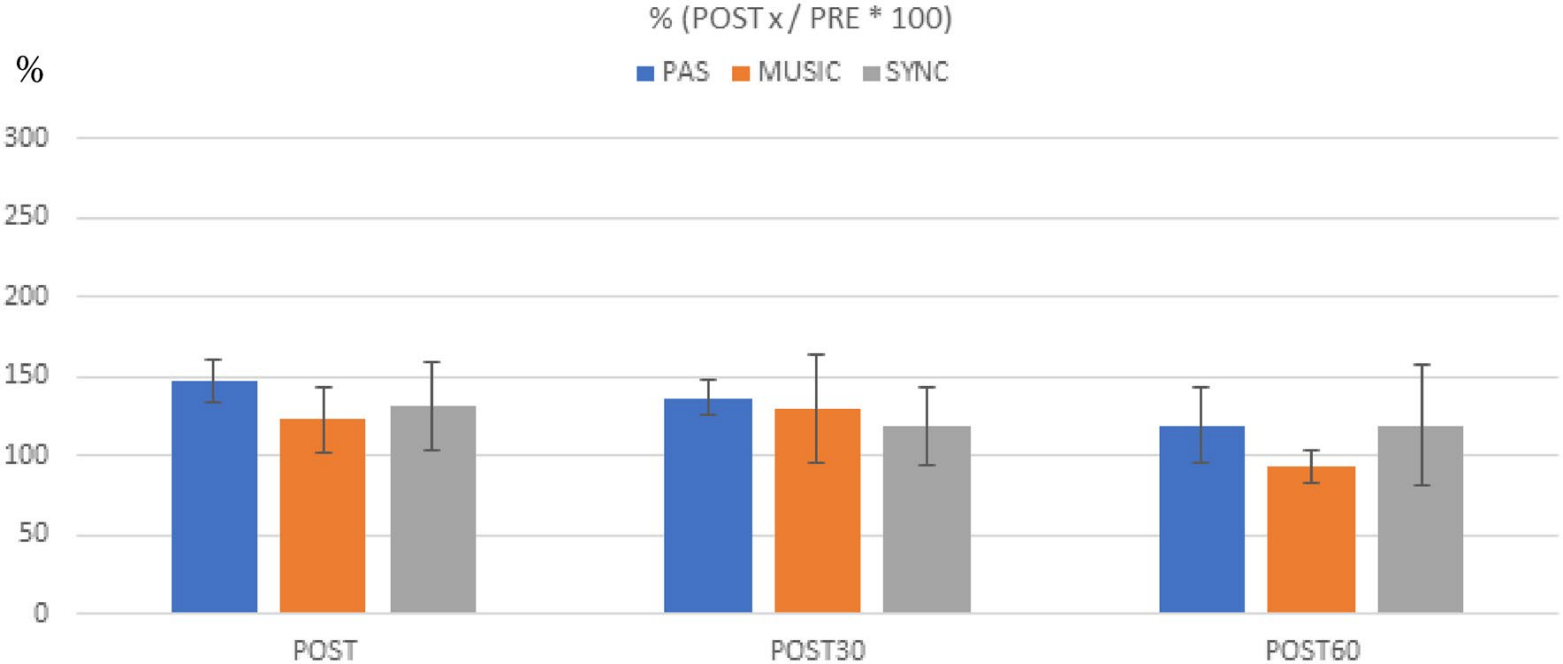
SCI patient MEP amplitude change vs PRE (100%) and standard errors. None of these reached statistical significance.

**Figure 6:**
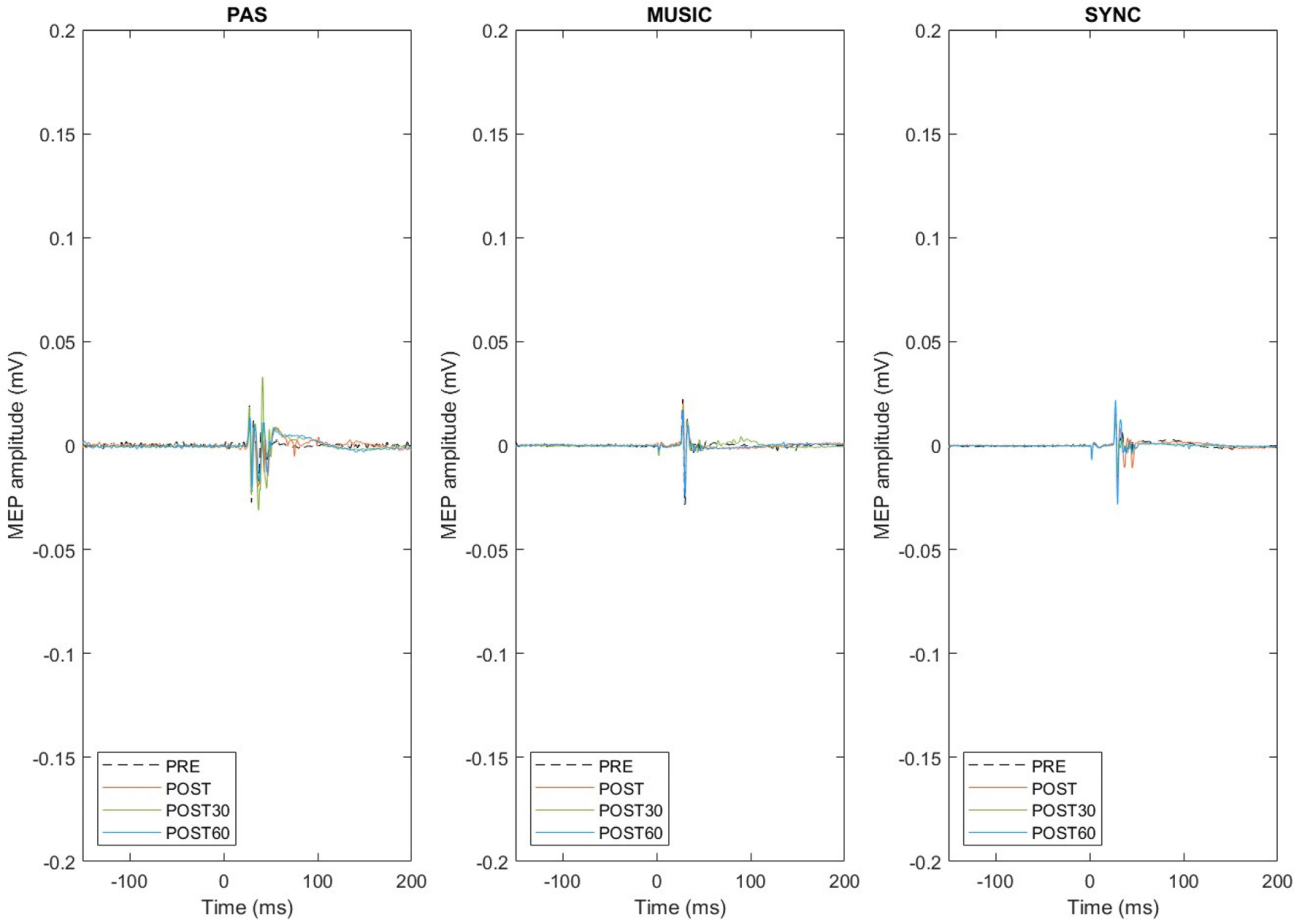
Representative MEP traces from one SCI patient (number 3). Each figure presents one session, each trace represents an average of 30 MEPs. Detrend function was used to bring pre stimulation EMG baseline to 0mV and 50Hz noise was filtered with a notch filter. Number 0 on x-axis (time) describes the time of given TMS pulse.

In within-session Friedman’s tests, there was a significant change in the MEP amplitudes across the four measurement points in PAS only [Χ^2^(3) = 8.10, *p* = 0.044] but not in PAS with self-selected music [Χ^2^(3) = 0.75, *p* = 0.861] or PAS with synchronized music [Χ^2^(3) = 2.25, *p* = 0.522]. In PAS only, post hoc testing (Dunn-Bonferroni) showed a trend towards MEP amplitude increase from PRE to POST and PRE to POST30, but this did not quite reach the Bonferroni-corrected significance level (*p* = 0.022 in both). In between-session Friedman’s tests, the MEP amplitudes did not differ significantly between the three sessions at baseline (PRE) [Χ^2^(2) = 0.25, *p* = 0.882] or from baseline to the post-stimulation time points [PRE-POST: Χ^2^(2) = 3.25, *p* = 0.197; PRE-POST30: Χ^2^(2) = 1.00, *p* = 0.607; PRE-POST60: Χ^2^(2) = 1.00, *p* = 0.607]. Together, these results suggest that in SCI patients, the PAS only protocol increased MEP amplitudes whereas the music PAS protocols did not yield statistically significant effects on MEPs (Fig. 5). Original MEP traces from one representative SCI patient (Patient 3) are presented in Fig. 6. In this patient, increase in MEPs after PAS can be seen in PAS only (orange, red and blue line compared to black, dotted line) but not in other sessions. Background noise did not exceed 50µV in any patient. MEP results of all patients as absolute values (µV) and percents (% POSTx/PRE*100) are presented in Supplementary information 1b.

**Figure 7:**
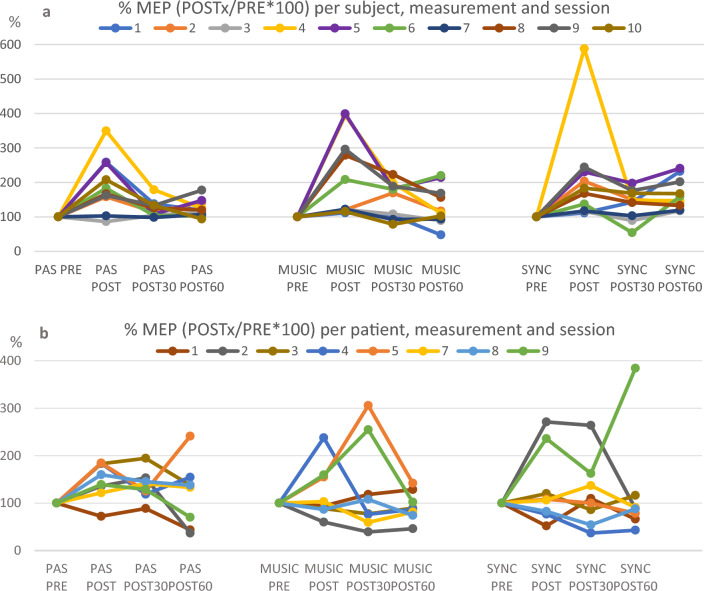
Individual variation of MEPs as % in healthy subjects (5a) and SCI patients (5b) per measurement and session.

Patients showed remarkable within-subject and between-subject variability, which was larger in both sessions with music than in PAS only (Fig. 7b). Variability can also be seen in healthy subjects, especially in the session with self-selected music (Fig. 7a). However, there was a trend in both groups: in healthy subjects MEP potentiation was prolonged in session with synchronized music and in SCI patients it was stronger without music.

### Subjective alertness and comfort in SCI patients

Subjective alertness and comfort scores of SCI patients are shown in Table 3. In Friedman’s tests, there were no significant differences between the three sessions in alertness before the stimulation [Χ^2^(2) = 0.42, *p* = 0.810] or in comfort during the stimulation [Χ^2^(2) = 2.00, *p* = 0.368]. In alertness after the stimulation, there was a marginally significant difference between the sessions [Χ^2^(2) = 5.77, *p* = 0.056]. Alertness appeared to be slightly higher after the PAS with self-selected music than after PAS only, but this did not reach statistical significance (*p* = 0.099).

**Table 3:** SCI patients (n=9) averaged alertness and feelings on scale 1–5 (alertness: 1 = very alert, 5 = very tired, feelings: 1 = very comfortable, 5 = very uncomfortable).

Session	Alertness PRE	Alertness DURING	Feelings DURING
PAS only	2.0 ± 0.4	2.56 ± 0.4	2.0 ± 0.3
PAS + own music	2.22 ± 0.4	1.89 ± 0.3	1.78 ± 0.2
PAS + sync music	1.89 ± 0.3	2.22 ± 0.3	1.78 ± 0.3

SCI patients gave more verbal feedback than the healthy subjects. Three patients reported that the PAS stimulation felt shorter with any type of music and 2 reported that self-selected music put them in a good mood. Two patients also reported that they felt less tired during the PAS stimulation and 2 other patients reported that the stimulation felt less uncomfortable with music than without music. Negative feedback about the music concerned motor imagery; 2 patients felt it was more difficult to focus on thinking about the muscle of interest when listening to music. Synchronized music evoked positive and negative feelings; 2 patients did not like it, others described it as monotonous but suitable for stimulation or “listenable” or “not bad”.

## Discussion

This study sought to determine if the effect of high-PAS can be augmented with inclusion of music, which was either temporally synchronized or not synchronized to the timing of the TMS, in terms of muscle motor responses and subjective alertness and comfort during stimulation. In healthy subjects, all three conditions (PAS, MUSIC, and SYNC) increased MEP amplitudes, but the effects of the stimulation remained stronger 60 min after the stimulation when music synchronized to TMS pulses was used. However, a similar effect was not found in SCI patients. Overall, the statistical significance of the effects was weaker in SCI patients due to expected greater within-subject and between-subject variability. However, in SCI patients the increase in MEP amplitudes was more stable and predictable without music.

There are many explanations for the possible strengthening or lengthening effect of synchronized music on high-PAS in healthy subjects. Since the effect of PAS is thought to be based on LTP-like mechanisms^8^, it is plausible that POST values represent early LTP effects (which start to decay at POST30), whereas POST 60 values reflect late LTP effects^55^. Although early-phase LTP does not require protein synthesis, late-phase LTP is dependent on it and on the activation of transcription factors. In late-phase LTP, the structural changes are evident and the effect can last at least 24 h.^55^ Chen et al.^31^ suggest there is a link between auditory and motor systems in the context of rhythm. Listening to music associated with movement can activate premotor cortex^29,31^ and increase corticospinal excitability^41^ and motor imagery causes the same type of effect on secondary motor cortex^56^. These factors could together increase MEP potentiation. The repetitive beat of synchronized music can help participants predict the timing of incoming PAS stimulus, which can be understood within the general framework of predictive coding. Neural responses are modified by expectations and these expectations are hierarchically organized and require interactions between distinct brain areas.^57,58^ Predictable auditory information can activate motor representations in an anticipatory muscle-specific manner, even in the absence of intention to move^43^. It is possible that these sound-related, automatically prepared potentials caused by repeated melody predicting the incoming stimulus can lower the stimulation threshold and strengthen the effect of high-PAS. When the tempo and beat of music are synchronized with TMS pulses and pulses are easier to predict, it may also help participants to avoid unpleasant sensations and to keep up with the stimulation rhythm and motor imagery. Interestingly, negative emotions or irritation evoked by synchronized music did not seem to affect the results.

Although self-selected music did not significantly increase or decrease the effect of high-PAS in healthy subjects, it may have had an inhibitory effect in SCI patients in some cases. Individuals seem to react differently to music during high-PAS. The reason for the individual variation is not known and warrants further research. Clinically, it would be important to identify what type of music is most beneficial and who benefits from music and who does not. The style or genre of the music did not seem to affect to the results, but tempo and beat may potentially have some effect. To control the style of the music, we collected a list of songs every participant listened to during the stimulation session and categorized them into five different genres. However, categorizing by rhythm and tempo was not possible because the participants listened to multiple songs with a variety of tempos during the 20-min stimulation; this is indeed a limitation of this study and an important subject for future studies. Listening to metrically strong rhythms or high-groove music can enhance corticospinal excitability and music associated with movement^41,59^. Medium to fast tempo music can reduce fatigue in monotonous activities but fast tempo may impair attention after a certain time. Slow music can temporarily help maintain attention but could decrease the level of alertness and attention over a long period of listening^60^. Therefore, the tempo of the participant’s selected music may have positive or negative effects on high-PAS by affecting attention and alertness, which are both significant factors in SCI patients compared with healthy individuals. It would be interesting to clarify these potential effects in connection with our clinical stimulation setup. The participant’s musical skills or attitude towards music may be other possible explanations for individual variation, as previous studies have shown higher PAS-induced MEP amplitudes in musicians^47,48^. Although no participants were professional musicians or actively practiced any musical activities, some had a history of playing an instrument, singing, or dancing. A more detailed description of musical relations would have been required to analyse the correlation between musicality and MEP increase. Repeating the setup of this study with musicians would clarify how musical background affects the results.

In some cases, music in general seemed to have a negative, distracting effect on high-PAS. Some SCI patients reported difficulties in focusing on motor imagery when listening to music, especially when it was self-selected. It is plausible that self-selected music may direct attention to itself and bring emotions and personal memories associated with the music to mind, which can disturb the focus on the motor imagery. Although motor imagery is not required for MEP amplitude potentiation in healthy individuals^17^, it can be beneficial in SCI patients due to decrease of RMTs (which are in generally higher in these patients) and secondary motor cortex activation^56^. Overall, this study supports the idea that attention can play an important role both in conventional PAS protocols^11^ and high-PAS in SCI patients. We intentionally did not place any restrictions on the self-selected music and the participants were instructed to choose music they like. According to Brattico et al.^61^, happy or sad music do not significantly differ in the state of brain activity in the motor cortex. The participants had the possibility to describe their feelings after the stimulation and all comments related to self-selected music were positive.

Other types of additional stimuli may affect the outcome of high-PAS. According to Lepping et al.^62^, the motor cortex activates more during musical stimuli compared with non-musical stimuli. It is noteworthy that the TMS device itself causes mechanical noise, namely the steady buzzing of the fan and strong click noise of the coil discharge. Although all participants used hearing protection due to click noise, this did not prevent them from hearing it either during the control session or during the sessions with music. The click sound alone can evoke long-latency responses in pontomedullary reticular formation, possibly through the acoustic startle circuit or vestibulo-reticulo-spinal pathway^39^. Therefore, additional non-musical auditory stimulus is always present in our high-PAS protocol and the aim of this study was to compare it with two different musical stimuli. Combining high-PAS with other auditory or visual stimuli remains as an important topic for future research.

SCI patients were on average older than healthy subjects, and almost all of them used CNS-active medication. This makes them more prone to fatigue and their attention is more easily disturbed; this is one of the plausible explanations why the positive effect of synchronized music was observed in healthy subjects but not in patients. As self-selected music does not strengthen the effect of high-PAS and can potentially have a distracting effect in some patients, there is no reason to recommend music routinely as part of a high-PAS protocol. However, pleasant music seems to increase alertness during stimulation especially in SCI patients. In the clinical setting, listening to self-selected music may be helpful for patients who become tired or fall asleep easily during stimulations.

The AH muscle has been a standard muscle of choice in our healthy subject experiments due to lower intrinsic variability of the MEPs. However, we selected the APB muscle of the hand for our SCI patients with incomplete tetraplegia, as the lower limb muscles were too weak or too spastic for the experiments for many of them. Although greater intersubject variability observed in SCI patients than in healthy subjects may be partly explained by the involvement of the upper vs lower limb, many other factors in the patient population, such as greater burden of other diseases, medication, and older age, may also play a role. It is unlikely that muscle choice affected the polarity of the observed changes in MEPs. In our previous work, we have stimulated different nerves in healthy individuals^18,49^ and in spinal cord injury patients^11^ and obtained similar and stable results regardless of the selected nerves. We calculated the ISIs and peripheral stimulation intensities individually for each subject, therefore minimizing the effect of choice of the motor cortical sites, nerves, and muscles^17^.

The heterogeneity and limited number of suitable participants are always a challenge when studying SCI patients. Although most of the patients participating in this study had relatively mild injuries, their functional and cognitive abilities and potential receptivity to high-PAS varied due to age, medication, and greater disease burden. Importantly, MEP evaluation can also be compromised in SCI patients due to variability in spasticity. The standard procedure of measuring an average of 30 MEPs per measurement reduces the impact of intrinsic MEP variability. Other indicators for positive effects of high-PAS, such as manual muscle testing (MMT) or functional tests^11^, may be useful in future studies. Importantly, these data show that the results from healthy subjects are not always directly generalizable to clinical patient groups, although they likely provide information on how a treatment would have affected the patients if confounding factors such as CNS-active medications were not present.

CNS-active medications are frequently used to manage spasticity and neuropathic pain, which are very common problems after SCI, and many of these medications work through the GABAergic system. For example, the GABA B receptor agonist baclofen is an effective drug for spasticity^16^. In our study, 5/9 SCI patients received on-going baclofen or other GABA agonist medication. Discontinuation of these medications for the duration of the study was not possible because it would have had a negative impact on functionality and well-being of the patients. The exact cellular and molecular level mechanism of high-PAS action is still under investigation^11^, hence the role of GABAergic system on high-PAS is also not known. It is possible that the medication affected the results and accounts at least partly for the differences that we observed between patients and healthy subjects. For example, baclofen can result in significant changes in long- and short-interval intracortical inhibition (LICI and SICI, respectively)^63^ and reduce intracortical signal propagation^64^, even though it has no effect on motor thresholds or MEP amplitudes^63^. Baclofen has been shown to decrease short-term potentiation (STP) in vitro^65^ as well as PAS- induced LTP-like plasticity in human motor cortex^66^, and it might have contributed here to the lack of long-term effect in SCI patients that was seen at POST60 in healthy subjects. Here the number of patients was too small to elucidate the effects of each individual drug or the timing of administration of medication in each patient, as each patient had a unique combination of drugs and their doses. In other studies where we have administered high-PAS to study its effect on motor improvement, we did not interfere with other rehabilitation and medication received by patients. Although most of these patients received medications that are typical for this patient group, the beneficial effect of high-PAS was not abolished^11^. This adds to the clinical feasibility of these results, as in clinical work it is not always possible to control for exact administration timing of medication and other therapies.

In conclusion, to our knowledge this was the first study to report on the effect of music on PAS experiments in the motor system. Although in healthy subjects music does not abolish the effect of high-PAS and synchronized music can even prolong the positive effects on MEPs, in incomplete SCI patients music seems to increase the variability of outcomes and thus cannot be routinely recommended as part of future PAS-treatments for incomplete SCI. However, the effect of music seems to be individual, and music did not abolish the effect of high-PAS in all patients. In the future, studies with larger patient samples and more systematic evaluation of the mediating effects of clinical and music background factors are needed to determine the clinical applicability of music in PAS treatment. In clinical settings, the possible detrimental effects of falling asleep during stimulation, which is especially relevant in patients who are heavily medicated, should be weighed against the possible distracting effect of music. MEP recordings after single sessions may assist in guiding these decisions and in the longer term combining them with manual muscle testing and other functional tests would give valuable, clinical information about the relationship of music and high-PAS. More generally, these results highlight the importance of understanding the effects of any additional stimuli added to neuroplasticity-promoting protocols that have shown therapeutic effect in laboratory setting when bringing these protocols to real clinical practice and adjusting them to patents’ individual needs.

### Supplementary Information

Supplementary Information 1.

Supplementary Audio 1.

## Data Availability

The datasets generated during and/or analysed during the current study are available from the corresponding author on reasonable request.
