# Supplementary material for: The effects of music combined to paired associative stimulation on motor-evoked potentials and alertness in spinal cord injury patients and healthy subjects
Source: Sci Rep. 2024 May 3;14:10194. doi: 10.1038/s41598-024-60984-w (PMC11068768; doi:10.1038/s41598-024-60984-w)
Supplement: Supplementary file 1 — Supplementary Information 1. [file 41598_2024_60984_MOESM1_ESM.pdf]

Supplementary information 1a. MEP results of the healthy subjects as absolute values and percentages.

Healthy subjects Absolute value MEPs (microvolts)

Measurement	Subject	PAS	MUSIC	SYNC
**PRE**	1	464,3333	228,643	306,433
	2	137,7586	186,4667	140,6333
	3	446,6333	337,931	472,2
	4	445,833	1255,533	477,567
	5	275,2	241,9286	614,3333
	6	480,8	259,0333	337
	7	2183,467	2263,267	1560,793
	8	879,4667	637,5	1135,533
	9	298,2333	250,2	189,6207
	10	145	180,4828	181,6667
	**mean**	**575,6725**	**584,0985**	**541,578**
	**STD**	**603,3889**	**676,2216**	**461,5311**
	**STE**	**190,8083**	**213,8401**	**145,9489**
**POST**	1	1201,233	255,167	341,6
	2	218,1034	225,1333	286,1
	3	385,3	409,4333	546,2333
	4	1558,5	4953,069	2807,533
	5	707,6786	965,3333	1415,033
	6	881,0667	539,4	462,5667
	7	2245,4	2761,6	1828,133
	8	1466,933	1776,6	1907,069
	9	484,129	741	463,2667
	10	301,1667	208,5	332,963
	**mean**	**944,9511**	**1283,524**	**1039,05**
	**STD**	**661,183**	**1525,346**	**888,4232**
	**STE**	**209,0844**	**482,3566**	**280,9441**
**POST30**	1	639,4	237,467	436,8
	2	157,5667	315,7333	210,9667
	3	466,2667	365,1	422,6667
	4	796,2	2492,167	707,0667
	5	296,9333	440,6207	1214,4
	6	502,5	465,4667	182,7667
	7	2149,933	2091,567	1609,4
	8	1105,633	1421,552	1605,033
	9	396,6667	472,2069	333,9333
	10	193,7931	141,5517	306
	**mean**	**670,4893**	**844,3432**	**702,9033**
	**STD**	**593,4507**	**844,617**	**563,0058**
	**STE**	**187,6656**	**267,0914**	**178,0381**
**POST60**	1	553,1	109,3793	710,4
	2	195,1667	218,1667	201,7667
	3	506,8	301,3333	555,1333
	4	557,4	1395,1	700,7
	5	405,9333	517,8333	1478,414
	6	490,3667	569,6667	534,7667
	7	2330,933	2117,233	1862,067
	8	1055,267	995,5	1511,167
	9	529,1379	421,0333	382,2667
	10	136,0333	185,0741	303,913
	**mean**	**676,0138**	**683,032**	**824,0594**
	**STD**	**631,7678**	**641,9787**	**578,7807**
	**STE**	**199,7825**	**203,0115**	**183,0265**

Healthy subjects

Data in % normalized to pre-PAS (POSTx/PRE*100)

Measurement	Subject	PAS	MUSIC	SYNC
POST	1	258,7006	111,6006	111,4762
	2	158,3229	120,7365	203,4368
	3	86,26763	121,1588	115,6784
	4	349,5704	394,4993	587,8825
	5	257,1506	399,0158	230,3364
	6	183,2501	208,2357	137,2601
	7	102,8365	122,0183	117,1285
	8	166,7981	278,6824	167,9448
	9	162,3323	296,1631	244,3123
	10	207,7011	115,5235	183,2824
	**mean**	**193,293**	**216,7634**	**209,8738**
	**STD**	**78,60399**	**117,2195**	**141,1977**
	**STE**	**24,85676**	**37,06807**	**44,65064**
POST30	1	137,7028	103,8593	142,5434
	2	114,3788	169,3243	150,0119
	3	104,3959	108,0398	89,51009
	4	178,587	198,4947	148,056
	5	107,8973	182,1284	197,6777
	6	104,5133	179,6937	54,23343
	7	98,46422	92,41362	103,1142
	8	125,7163	222,9885	141,3462
	9	133,0055	188,7318	176,106
	10	133,6504	78,4295	168,4404
	**mean**	**123,8312**	**152,4104**	**137,1039**
	**STD**	**23,83368**	**51,35567**	**43,1978**
	**STE**	**7,536871**	**16,24009**	**13,66034**
POST60	1	119,117	47,83846	231,8288
	2	141,6729	117,0004	143,47
	3	113,4712	89,17007	117,5632
	4	125,0244	111,1162	146,7229
	5	147,5048	214,0439	240,6534
	6	101,9897	219,9202	158,6845
	7	106,7538	93,54767	119,3026
	8	119,9894	156,1569	133,0799
	9	177,4241	168,2787	201,5954
	10	93,81609	102,5439	167,2916
	**mean**	**124,6763**	**131,9616**	**166,0192**
	**STD**	**24,85326**	**56,06126**	**44,37027**
	**STE**	**7,859291**	**17,72813**	**14,03111**

Supplementary information 1b. MEP results of the SCI patients as absolute values and percentages.

SCI patients Absolute value MEPs (microvolts)

Measurement	Patient	PAS	MUSIC	SYNC
**PRE**	1	81,53571	79,56667	169,871
	2	445,6667	916,7333	326,9333
	3	94,96667	86,43333	79,29032
	4	52	87,25	176,6667
	5	156	164,2667	1141,533
	7	689,7241	730,8571	478,7778
	8	191,1818	380,9412	235,0909
	9	101,8667	53,53333	60,4
	**mean**	**226,6177**	**312,4477**	**333,5704**
	**STD**	**224,6036**	**335,9301**	**353,4177**
	**STE**	**71,0259**	**106,2304**	**111,7605**
**POST**	1	58,82143	74,7931	88
	2	605,1	549,5	886,3793
	3	173,8333	76,34483	95,16667
	4	95,6	207,3448	135,4444
	5	287,9333	254,5333	1235,333
	7	839,1	753,9231	508,8333
	8	305,64	330,0417	194,1071
	9	141,6	85,36667	142,5333
	**mean**	**313,4535**	**291,4809**	**410,7246**
	**STD**	**273,4435**	**246,7149**	**433,2945**
	**STE**	**86,47042**	**78,01811**	**137,0197**
**POST30**	1	72,28571	94,1	186,2667
	2	682,6667	359,9667	862,6667
	3	184,6333	66,93333	68,17241
	4	61,76667	66,58621	65,10714
	5	195,5333	502	1141,133
	7	971,7143	434	655,7333
	8	277,6923	410,7143	127,2222
	9	131,25	136,3333	98,11111
	**mean**	**322,1928**	**258,8292**	**400,5516**
	**STD**	**328,5247**	**184,7922**	**424,6386**
	**STE**	**103,8886**	**58,43642**	**134,2825**
**POST60**	1	35,48276	102,2333	112,5333
	2	163,8	422,3571	288,9
	3	130,8667	77,12903	92,2
	4	80,34483	75,6	75,74074
	5	376,2	233,1	894,0667
	7	917,4444	592,4	434,2667
	8	263,1724	282,069	206,7742
	9	71,37931	54,62069	232,1379
	**mean**	**254,8363**	**229,9386**	**292,0774**
	**STD**	**290,2521**	**194,6964**	**270,5753**
	**STE**	**91,78577**	**61,56842**	**85,56342**

N=8, patient 6 excluded from the data

SCI patients

Data in % normalized to pre-PAS (POSTx/PRE*100)

Measurement	Patient	PAS	MUSIC	SYNC
POST	1	72,14192	94,00055	51,80403
	2	135,7741	59,9411	271,1193
	3	183,0467	88,32799	120,0231
	4	183,8462	237,6445	76,66667
	5	184,5726	154,9513	108,217
	7	121,6573	103,156	106,2776
	8	159,8688	86,63849	82,56684
	9	139,0052	159,4645	235,9823
	**mean**	**147,4891**	**123,0156**	**131,5821**
	**STD**	**39,03902**	**57,60629**	**78,80593**
	**STE**	**12,34522**	**18,21671**	**24,92062**
POST30	1	88,65528	118,2656	109,6519
	2	153,1788	39,26624	263,8662
	3	194,4191	77,43926	85,97823
	4	118,7821	76,31657	36,8531
	5	125,3419	305,6006	99,96496
	7	140,8845	59,38233	136,9599
	8	145,2504	107,8157	54,11618
	9	128,8449	254,67	162,4356
	**mean**	**136,9196**	**129,8445**	**118,7283**
	**STD**	**30,50371**	**97,00652**	**71,40731**
	**STE**	**9,64612**	**30,67616**	**22,58097**
POST60	1	43,51806	128,4876	66,24636
	2	36,75393	46,07197	88,36664
	3	137,8027	89,23529	116,2815
	4	154,5093	86,64756	42,87212
	5	241,1538	141,9034	78,32156
	7	133,0161	81,05551	90,70318
	8	137,6556	74,04528	87,95499
	9	70,07131	102,0312	384,3343
	**mean**	**119,3101**	**93,68473**	**119,3851**
	**STD**	**67,54436**	**30,44303**	**109,1163**
	**STE**	**21,3594**	**9,626931**	**34,50559**

N=8, patient 6 excluded from the data

Supplementary information 2 / Alertness and comfort questionnaire

Before stimulation

How did you sleep last night?

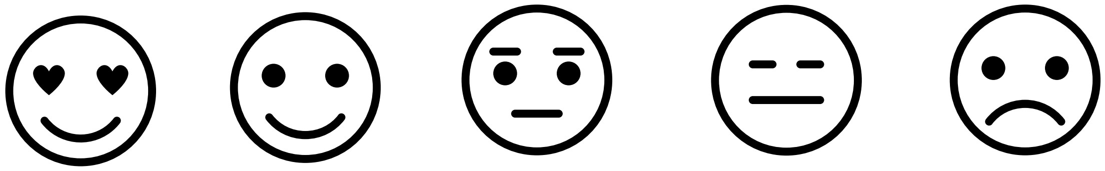

Very well Very badly

How would you describe your state of alertness at the moment?

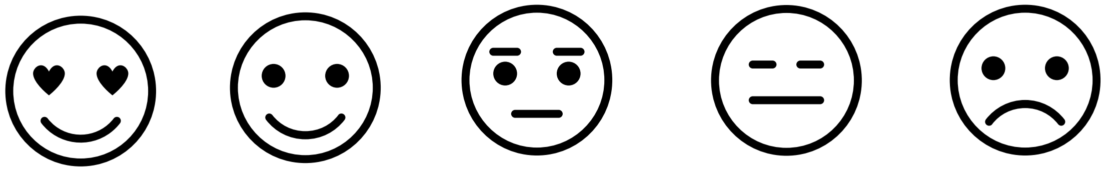

Very alert Very tired

After stimulation

How would you describe your state of alertness during the stimulation?

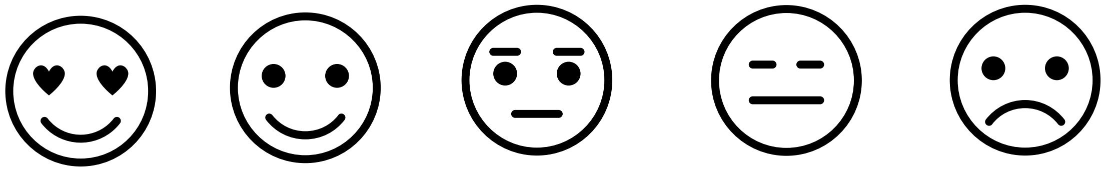

Very alert Very tired

How would you describe the feeling caused by stimulation?

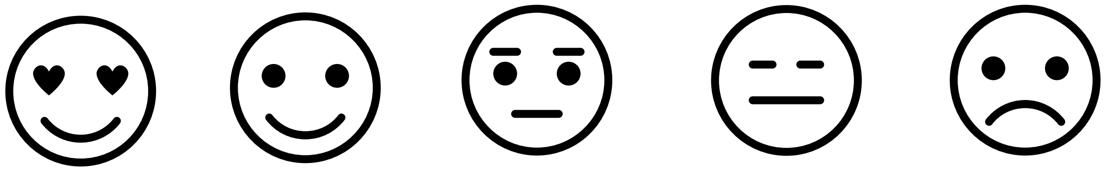

Very comfortable Very uncomfortable
